# Supplementary material for: Are carbon emissions trading and green financial instruments synergistic? -Comprehensive quantitative research based on content analysis
Source: PLoS One. 2024 Mar 7;19(3):e0298601. doi: 10.1371/journal.pone.0298601 (PMC10919601; doi:10.1371/journal.pone.0298601)
Supplement: S1 Data — (ZIP) [file pone.0298601.s001.zip › Data/地级市Z_F工作报告.zip!/╜¡╬≈/╔╧╚─╩╨/└┤╫╘╣1⁄2╓┌║┼í╛┬φ┐╦╩2╛▌═°í┐_2012.docx]

各位代表：

现在，我代表市人民政府，向大会报告政府工作，请予审议，并请市政协委员和列席会议的同志提出意见。

一、2012年工作回顾

刚刚过去的2012年，是新世纪以来我国经济发展最为困难的一年。面对复杂严峻的国际形势和改革发展稳定的繁重任务，在省委、省政府和市委的坚强领导下，我们牢牢把握主题、主线和稳中求进的总基调，紧紧围绕建设宜居宜业宜游现代化区域中心城市的奋斗目标，审时度势，攻坚克难，召开六个“千人大会”，奋力推进“八大战略”，全力化解经济下行压力，圆满地完成了市三届人大二次会议确定的目标任务，改革开放和现代化建设取得新的成绩。

一年来，我们主要做了以下工作：

（一）着力提升发展质量和速度，综合经济实力进一步增强

主要经济指标超过全省平均增幅。预计全市生产总值1306亿元，增长13.0%，增幅高于全省2.3个百分点；固定资产投资1080亿元，增长30%，增幅与全省持平；财政总收入200.36亿元，增长32.2%，增幅高于全省5.9个百分点，县县财政收入过9亿元，新增鄱阳县、万年县财政收入过10亿元；社会消费品零售总额439亿元，增长16％，增幅高于全省0.5个百分点。

发展的质量和效益持续提升。三次产业的比重调整为14.9∶54.4∶30.7，第二、第三产业比重均比上年提高了0.5个百分点；全部工业增加值占生产总值的比重达到46.7%，比上年提高1.7个百分点。财政总收入占生产总值的比重达到15.4%，比上年提高1.8个百分点。城镇居民人均可支配收入突破2万元，达到20170元，增长14%；农民人均纯收入突破7000元，增长14.1 %。

（二）着力发展实体经济和保护生态，可持续发展能力进一步提升

新型工业化快速发展。全年开工、在建投资亿元以上工业项目130个，总投资583亿元，工业投资占全部固定资产投资的比重比上年提高7.3个百分点，达到54%。新增工业园区开发面积10.8平方公里，广丰、玉山工业园被省政府认定为重点省级工业园区，横峰工业园升级为横峰经济开发区。新增规模以上工业企业50家，总户数达575户。新增主营业务收入超亿元企业10户，其中过10亿元企业新增5户，过50亿元企业新增1户。预计规模以上工业企业实现主营业务收入1750亿元，增长20%，实现工业增加值380亿元，增长20%；工业园区实现主营业务收入1800亿元、增长22%。有色金属、新能源、机电光学、新型建材四大主导产业占规模以上工业增加值比重继续提升，达到75%，提高2.7个百分点。特别是重点扶持的战略性新兴产业快速发展，实现增加值170亿元，增长23.6%，晶科能源被普华永道会计事务所评定为2012年全球最具可持续发展的4家光伏企业之一。

农业现代化迈出新步伐。主要农产品产量持续获得丰收，粮食产量连续九年增长，达338万吨，增长4.7%；预计粮食、水产、蔬菜、生猪、节粮型畜禽、苗木花卉、油茶、水果等10个优势特色产业综合产值可达406.9亿元。农业科技创新取得新突破，累计选育农作物品种82个、地方畜禽品种36个，其中“赣晚糯6号”成为目前世界上第一个富含天然维生素A的特种水稻品种。农业机械化逐步加快，预计全市农机总动力可达618万千瓦，同比增长10%；水稻耕种收综合机械化率达到61.9%，同比提高3.3个百分点。农业信息化进程持续提升。农业产业化迅速发展，新增规模以上农业龙头企业57家，国家级龙头企业增至4家，省级以上龙头企业增至84家，市级龙头企业增至181家，预计市级以上龙头企业可实现销售收入220亿元，增长27%，带动农户120万户，促进农民增收24.5亿元。农业现代化经营形式继续优化，新增农民专业合作社523家，总数达到2619家。农业生产区域化取得重要成果，婺源县成功创建国家有机产品认证示范区，新增铅山、广丰、上饶县为省级农业示范县。

现代服务业快速增长。旅游业保持强劲发展势头。三清山被联合国教科文组织正式列入世界地质公园名录，婺源县被国家旅游局授予首批“全国旅游标准化示范县”，婺源国家乡村旅游度假实验区正式揭牌，万年神农源、德兴大茅山景区成功获批国家级风景名胜区。预计全年接待游客突破4000万人次、增长37.2%，旅游总收入达到300亿元、增长39.3%，旅游人次、旅游总收入和门票收入三项指标将继续保持全省第一，其中鄱阳湖湿地公园接待游客增幅继续领先全省，上饶旅游知名度在全国100个旅游设区市排名由上年的第87位升至第47位。金融保险业快速发展。交通银行、民生银行、浦发银行上饶分行和9家商业银行一级支行顺利开业。信州区农信社、广丰县农合行、婺源县农信社改制组建农村商业银行工作稳步推进。新设立小额贷款公司5家、融资性担保公司3家。全市银行业金融机构人民币存款余额1405.5亿元，较年初增加239.9亿元，增长20.6%；商业银行贷款余额827.8亿元，较年初增加136.6亿元，增长19.6%。全市保险业金融机构发展到25家，保险业总保费27.26亿元。政策性农业保险健康发展。鑫星农牧上市已报中国证监会待批。商贸流通业繁荣活跃。信州区、婺源县获批省级服务业综合改革试点县（区），国家农产品现代物流综合试点建设扎实推进，全市新增交易额超亿元的市场6家，总数达到46家。全国第三批再生资源回收体系建设试点工作顺利启动。

生态环境进一步优化。完成造林41.7万亩，省级“森林十创”先进单位达39家。全市主要河流监测断面水质达标率99.1%，城市环境空气质量全部在二级以上，削减化学需氧量5320吨、二氧化硫1650吨、氮氧化物1100吨、氨氮840吨，单位生产总值能耗下降5.2%，单位工业增加值能耗下降2.1%。

（三）着力增强发展动力和后劲，发展基础进一步夯实

基础设施建设扎实推进。三清山机场奠基开工。杭长高铁、合福高铁加快推进。德上高速竣工通车，新增通车里程61.2公里，境内高速总里程达到617公里。德上高速三清山和怀玉山连接线、沪昆高速上饶经开区互通及连接线开工建设。新增农村公路970公里，基本完成改渡建桥任务。国电黄金埠电厂二期工程获国家能源局核发“路条”，即将开工建设。天然气入饶项目中石油主管道全部完工，中心城市春节前正式通气。宁德港上饶码头列入福建省2013年重点工程即将开工。万年港综合码头基本建成。上饶移动开通4G网络，移动电话用户新增100万户、总数达480万户。互联网用户较上年翻番，达55万户。有线电视用户达68万户。完成水利投资23.3亿元，新增和改善灌溉面积 14.8万亩，新建高标准农田23.6万亩。

新型城镇化加快步伐。全市开工城建项目550个，完成投资260亿元，新增建成区面积30平方公里，城镇化率预计提高2个百分点，达到43.7%。中心城市开工城建项目103个，完成投资118亿元，上饶大桥、茶圣中路、天佑大道一期、三江城市综合体一期安置房、三江导排渠、城南大道等项目顺利建成，老火车站城市综合体、广信大厦、6000套保障性住房和5000套安置房、三清山机场大道、上饶师范迁建、城市饮水工程等项目建设进展加快。万年青建材商城、万达商业广场等大型商业综合体落户中心城市。创建省级生态园林城市、省级文明城市、省级森林城市工作深入扎实，中心城市共防共治违法违章建筑取得明显成效，城市形象进一步提升。新农村建设筹集资金10.9亿元，新建新农村村点1705个、农村清洁工程3746个，农村面貌有了新的改善。我市“突出三区四线，推动村镇联动”的新农村建设做法得到全省肯定。

各项改革顺利推进。农垦企业改革扫尾全面完成，七个系统改革工作受到省委、省政府表彰。朝阳磷矿政策性破产依法进入终结阶段。国有林场改革稳步推进。国有文艺院团转企改制任务全面完成。医药卫生体制改革进一步深化。企业一套表统计改革顺利实施。事业单位分类改革有序开展。中小学教师职称制度改革试点工作正式启动。万年县被列为全省县域经济发展先行先试试点县。

对外开放不断扩大。成功举办了央企走进鄱阳湖生态经济区、上饶籍在外创业成功人士返乡创业恳谈会、投资上饶（福建）恳谈会、投资上饶（香港）项目对接会等招商活动。与俄罗斯苏兹达里市缔结为国际友城。预计全年实际利用外资 6.6亿美元，增长10 %；引进省外5000万元以上项目资金 296亿元，增长19.7 %。兑现落实保持外贸稳定出口的政策措施，外贸出口保持平稳增长，完成出口总额25.5 亿美元，增长5.6 %。新增“走出去”企业2家。上饶至宁波北仑港“五定班列”运行平稳。南昌海关上饶办事处升格工作进展顺利。

全民创业深入推进。新增个体工商户25718户、私营企业4424户，分别增长3.4%、14.5%。非公经济完成增加值843亿元，增长15%。民间投资增长35.7%，对投资的贡献率达到84.7%。

（四）着力改善民生和维护稳定，民生社会事业进一步发展

民生工程扎实推进。全市财政用于教育、科技、文化、社会保障、保障性住房、农林水事务、医疗卫生、环境保护等重点民生领域的资金达到221.2亿元，占支出总量的74.4%，比上年提高2.3个百分点，保障了8大类89项民生工程任务全面或超额完成。新增城镇就业11万人、转移农村劳动力16万人；发放小额担保贷款10亿元，扶持带动就业4.1万人次。城镇职工养老、基本医疗、失业保险参保人数分别达到50.6万人、135万人和28.8万人，向62万60周岁以上老年人发放养老金1.9亿元，婺源县新型农村和城镇居民社会养老保险工作获得国务院表彰。城市低保、农村低保和城镇“三无”特困群众月平均保障标准分别提高到350元、170元和400元，农村五保户集中和分散供养月标准分别提高到220元和180元。参加新农合人数达555.4万人，参合率98.9%。新开工建设保障性住房39804套，发放廉租住房租赁补贴16500户，利用住房公积金贷款支持保障性住房建设试点工作正式启动。市残疾人康复和就业培训中心开工建设，新增残疾人就业基地63个，解决就业11132人。避灾移民搬迁5214人、深山区移民搬迁5044人。上饶县、广丰、铅山纳入原中央苏区振兴规划。解决了41.4万人饮水安全问题，完成农村危房改造15111户。

社会事业全面发展。义务教育和学前教育得到加强，职业教育健康推进，民办教育不断规范，万年县推进公办与民办教育协调发展的做法获得中央和省里肯定。在江西医学院上饶分院基础上申报设置江西医学高等专科学校工作接受省政府评估。国家二类城市语言文字工作评估顺利通过。科技创新工程稳步实施，国家级科技项目立项9项，信州区光学产业基地成功获批全省唯一的国家科技兴贸创新基地，圣达威电工“低损耗高频铅线圈新工艺开发与应用”成果荣获省科技进步一等奖。公共医疗卫生保障能力增强，县及以下医疗机构建设全面铺开，基层医疗卫生服务体系不断完善。贫困家庭重性精神病患者免费救治工作正式启动，累计免费治疗白内障手术1902例、白血病26例、先天性心脏病354例，收治尿毒症患者1504人。公共文化服务体系进一步完善，建设农家书屋2205家，文化信息资源共享工程全面覆盖县、乡、村。免费开放图书馆、文化馆、文化站，组织送戏下乡2000余场。全民健身和竞技体育取得新成绩，承办了省第四届全民健身运动会暨中华国际龙舟大赛、省第二届半程马拉松联谊赛等赛事，广丰县纪小飞获伦敦残奥会残疾人自行车场地团体竞速赛冠军。人口和计生工作进一步加强，低生育水平得到巩固，免费孕前优生健康检查全面启动，出生人口性别比下降4.1个基点。第八届村（居）“两委”换届选举工作圆满完成。

社会大局和谐稳定。加强重点行业和领域安全生产专项整治，安全生产形势总体平稳，实现了事故起数、死亡人数双下降。认真落实信访接待日、信访包案等制度，实行领导分类接访、分工包案、分片督导，健全了维护群众权益机制和社会矛盾常态化排查化解机制，群众到省、进京上访总量明显下降，确保了党的十八大等重大活动、重要节点的社会稳定。深入开展平安创建和突出治安问题整治活动，人民群众安全感进一步提升。民族宗教、涉台事务、气象水文、防震减灾、国防动员、人民防空、检验检疫、食品药品、妇女儿童、地方志、档案、保密、应急、工商、质监、石油、烟草、盐业、双拥、慈善等各项工作都取得新的成绩。

（五）着力优化环境和提升效能，政府自身建设进一步加强

法治建设扎实推进。严格按照法定权限和程序行使权力、履行职责，自觉接受市人大及其常委会的法律监督、工作监督和市政协的民主监督，广泛听取各方面的意见和建议，主动向人大常委会报告工作，认真接受人大代表询问和质询，按期办复人大代表建议190件、政协提案152件，满意率99.5%。

效能建设深入开展。加强政府网站、市长热线和在线访谈建设，深入开展“改进干部作风、优化发展环境、狠抓工作落实”和“集中整治影响发展环境的干部作风突出问题”活动，实施问责1722人次，各级政府服务发展、服务创业水平明显提高。

职能转变步伐加快。建立健全重大项目协调推进机制，精简市级非行政许可审批项目54项，精简率达35%，项目审批时间平均缩短37%；推进政务公开，主动公开信息13.2万条，进一步增强了政府工作的透明度。

廉政制度严格落实。强化行政监察和审计监督，推进惩治和预防腐败体系建设，认真开展工程领域、公务用车、公路“三乱”等专项治理，严肃查处了一批违法违纪案件，勤政廉政建设取得新成效。

各位代表，这些成绩的取得，是省委、省政府和市委统揽全局、正确领导的结果，是市人大、市政协有效监督、鼎力支持的结果，是全市人民同心同德、开拓进取、共同奋斗的结果。在此，我代表市人民政府，向付出辛勤劳动的全市广大干部群众，向大力支持政府工作的人大代表、政协委员、各民主党派、工商联、无党派、各人民团体和社会各界人士，向关心支持上饶建设的离退休老干部、老同志，向为上饶改革发展稳定作出积极贡献的驻饶部队、武警官兵和公安民警，向中央和省驻饶单位及所有关心支持上饶建设发展的同志们、朋友们，向在我市创业的投资者、建设者，致以崇高的敬意和衷心的感谢！

总结过去一年的工作，我们也清醒地看到，经济社会发展中还面临不少困难和问题。经济总量不大、产业层次不高、经济结构不优、中心城市辐射带动力不强、县域经济发展不平衡、城镇化水平低、人均水平低、农民人均纯收入低仍然是我们面临的主要矛盾，加快发展、加快转型仍然是当前的主要任务，加强和创新社会管理、保障安全、维护社会和谐稳定、改进工作作风、提高依法行政和工作效率、优化发展环境仍然是广大群众关心的切身利益问题。对这些困难和问题，我们将高度重视，继续采取有力措施，在今后工作中认真加以解决。

二、2013年主要任务

今年是全面贯彻落实十八大精神的开局之年，是实施“十二五”规划承前启后的关键一年，也是我市推进宜居宜业宜游现代化区域中心城市建设的重要一年。今年的发展仍然面临不少风险和挑战：国际经济形势依然错综复杂、充满变数，世界经济低速增长态势仍将延续，各种保护主义明显抬头，潜在通胀和资产泡沫的压力加大，世界经济已由危机前的快速发展期进入深度转型调整期。我国经济发展中不平衡、不协调、不可持续的问题依然突出，经济增长下行压力和产能相对过剩的矛盾有所加剧，企业生产经营成本上升和创新能力不足的问题并存，经济发展和资源环境的矛盾仍然突出。省内各设区市你追我赶的发展态势给我们带来的工作压力进一步加大。但我们也要看到发展的有利方面：我国经济社会发展基本面仍将长期趋好，国内市场潜力巨大，中央经济工作会议确定的宏观经济政策对中部地区崛起、鄱阳湖生态经济区建设和原中央苏区振兴发展提供了难得机遇和有力支撑。随着我市工业化和城镇化的快速发展，多年来高强度投入积累的能量逐步释放。特别是换届一年来，全市上下所形成的人心思干、人心思上、人心思和、求新思变的良好创业干事氛围，为我们加快发展提供了难得的工作基础。我们所处的时期仍然是大有作为、加快发展的重要时期，我们所面临的机遇是扩大内需、提高创新能力、促进经济发展方式转变的新机遇。只要我们始终保持清醒头脑，深刻理解、紧紧抓住、切实用好这样的有利条件和新机遇，因势利导，顺势而为，就一定能够战胜各种困难，在机遇和挑战中开创上饶发展的新局面。

今年政府工作的总体要求是：坚持以邓小平理论、“三个代表”重要思想、科学发展观为指导，深入学习和全面贯彻落实党的十八大精神和中央、全省经济工作会议精神，紧紧围绕建设宜居宜业宜游现代化区域中心城市的奋斗目标，继续深入推进“八大战略”，以提高经济增长质量和效益为中心，以项目建设、招商引资、决战园区为主要抓手，以投资拉动、消费促动、创新驱动、保增长、保民生、保稳定、促就业、促转型、促开放为工作着力点，开拓创新，扎实进取，努力实现上饶经济持续健康较快发展和社会和谐稳定。

今年经济社会发展的主要预期目标是：生产总值增长12%，财政总收入增长18%，固定资产投资增长25%以上，社会消费品零售总额增长15%，规模以上工业增加值增长15%，外贸出口增长5%，城镇居民人均可支配收入增长13%，农民人均纯收入增长13%，城镇化率提高2.5个百分点，人口自然增长率控制在7.52‰以内，城镇调查失业率控制在4.5%以内，居民消费价格涨幅及节能减排严格控制在省政府下达的指标之内。

实现上述目标，要重点抓好以下九个方面的工作：

（一）加快推进新型工业化，全力支持上饶经济技术开发区做大做强

坚定不移地实施主攻工业战略，围绕“三年翻一番”目标，加快调整产业结构，提高产业整体素质，力争全市规模以上工业增加值达到450亿元以上、增长15%以上，主营业务收入达到2300亿元、增长27%。

突出抓好一批重大工业项目建设。以战略性新兴产业、四大主导产业项目为重点，加快推进传统产业转型升级，突出抓好凤凰光学年产2.2亿套数码工作台和8800架普通生物显微镜、上饶胜科导线轴、广电高科技有限公司年产800MW太阳能电池生产线、中材机械有限公司年产3800台输送机、远翔实业年处理10万吨有色金属固体废物、中国汽车零部件基地、南方铜业改扩建、鑫赢公司年产5万吨环保精密铜棒、康盛实业纳米胶体电池、龚杏投资公司光学产业配套基地、江铜年产60万吨硫铁矿循环经济二期等131个列入省大中型工业项目计划的重点项目建设，力争凤凰光学、上饶胜科导线轴等20个重点项目年内建成投产。通过加大对工业的投资力度，力争实现工业投资占产业投资70%以上，四大主导产业占工业投资的70%以上，战略性新兴产业占四大主导产业投资的70%以上。通过大力推进战略性新兴产业发展，力争战略性新兴产业主营业务收入增长35%以上，占四大主导产业的比重达到50%以上。

全力支持工业园区做大做强。加强工业园区道路、通讯、供水、供电、供气、污染集控等基础设施建设，推进调园扩区工作，新增园区开发面积10平方公里以上。加强园区投入产出管理，提高入园项目的投资强度，县属园区入园项目投资强度要达到100万元/亩以上，上饶经济技术开发区要达到200万元/亩。加强园区产业配套建设，注重延长产业链，促进园区项目集聚、产业集群、形成特色。加强园区服务配套能力建设，完善金融保险、劳动中介、信息咨询、安全检查、产品检验、质量认证等综合服务功能。通过努力，力争全市园区主营业务收入实现2200亿元，主营业务收入过百亿元园区达到10个。

上饶经济技术开发区作为国家级经济技术开发区，是上饶实现工业化的主力，是上饶加快发展的希望所在。全力支持上饶经济技术开发区做大做强，是政府的重要职责。要通过“充分授权，封闭运行”，进一步完善园区的体制机制；要通过科学调整园区发展规划，加速产业集群；要通过加快大型物流配套能力建设，加速产业集聚；要通过招大引强，提升在全省经济技术开发区中的实力。力争通过三年的努力，使上饶经济技术开发区挤进全省工业园区三甲行列，园区工业主营业务收入、就业、工业投资三项指标占全市工业园区的三分之一，财政收入占市本级的三分之一。

积极推动工业化、信息化“两化”深度融合。完善信息基础设施，推进4G网络建设，发展现代信息技术产业体系，促进信息网络技术广泛运用。大力推进“数字上饶·无线城市”、宽带互联网、鄱阳湖生态经济区智慧工程、“三网融合”等信息化工程建设，打造“智慧上饶”，提高经济社会信息化服务水平。

切实帮助企业解决好生产、经营、建设中的困难和问题。进一步优化投资环境，严肃处理乱摊派、乱罚款、乱收费行为，坚决杜绝不作为、乱作为、推诿扯皮现象的发生，大力营造亲商、安商、富商的浓厚氛围。进一步强化主动跟踪服务措施，健全一对一帮扶、一站式办结、一单清收费、一条龙服务的“四个一”服务机制。进一步落实好招商引资的优惠政策，在项目申报、用地计划、供电供水、企业用工、交通运输、信用担保、融资等方面为客商提供帮助。进一步完善扶持小微企业的措施，支持小微企业搞活做强。进一步加大对重点企业的扶持力度，力争全年新增规模以上工业企业80家，新增主营业务收入过50亿元企业2家、过20亿元5家、过10亿元10家。

（二）加快推进农业现代化，全力支持鄱、余、万建设全国优质农产品种养加工基地

围绕建设现代农业强市目标，按照“保供增收惠民生、改革创新添活力”的要求，力促农业增效、农民增收、农村稳定。

多渠道促进农民增收。落实各项强农惠农富农政策，确保农民从政策性补贴中得到更多实惠。加强农村劳动力务工技能培训，有序引导农村富余劳动力外出务工增加工资性收入，计划培训农村劳动力7万人，新增转移农村劳动力7.2万人，农民人均务工收入增加600元以上。大力发展“农家乐”等庭院经济，鼓励引导农民多种经营，挖掘农业内部增收潜力，力争“农家乐” 发展到3万户以上。推动农村产权制度改革，稳步增加农民的红利、股金、租金等财产性收入。

大力提高农业综合生产能力。稳定粮食生产，提高优质稻比重，确保全年粮食播种面积稳定在900万亩左右，总产68亿斤以上。优化农业产业布局，大力发展优势特色农业，重点打造粮食、水产、果蔬、畜禽、油茶、木竹等六大百亿元产业。推进农业产业化经营，大力扶持农业龙头企业和农民专业合作组织发展，力争新增规模以上农业龙头企业85家，新增农民专业合作社400家以上，市级以上农业龙头企业年销售收入达到265亿元。

鄱阳、余干、万年农业资源丰富、农业特色明显、农业优势突出，具有建设全国优质农产品种养加基地的先天条件。今年，我们要把全力支持鄱、余、万建设全国优质农产品种养加基地作为推进农业强市建设的重点来抓。要大力扶持粮食、水产、畜禽等优势特色农业，加快建设一批专业化、标准化农产品生产基地；要大力引进和培育农业龙头企业，形成种养加一条龙、产供销一体化发展格局；要创新农业土地流转机制，促进耕地、林地、水面等生产要素向种养大户农业企业有序集中，形成规模经营；要完善农产品市场体系，加快粮食、水产品、畜禽产品等区域性产地专业市场建设，支持龙头企业建立健全农产品营销网络；要加大政策、项目扶持力度，确保资金、项目落实到位。力争三县今年粮食产量达到45亿斤、水产品总量达到30万吨、生猪出栏150万头，均占全市总量的三分之二左右。

不断加强农业基础设施建设。加快实施病险水库和水闸除险加固工程，力争全面完成大中型病险水库加固任务。继续实施鄱阳湖二期防洪工程、五河治理和中小河流治理项目、以及七一、饶丰和鄱湖灌区续建配套与节水改造项目。加快推进大坳、貊皮岭大型灌区改造项目的前期工作。力争建设高标准农田22.4万亩，新增、改善有效灌溉面积39.5万亩。加强农业气象防灾减灾和信息服务建设。

深入推进和谐秀美乡村建设。突出“三区四线"、村镇联动和村落连片，按照“五美四和谐"的要求，确保新建新农村村点1300个以上。深入实施农村清洁工程，大力推广农村垃圾处理“3+5”模式，推动垃圾分类无害化处理，保持农村干净整洁。加强农村社区建设，确保农村社会稳定、农民安居乐业。

（三）加快推进现代服务业发展，全力支持婺源、三清山建成全国一流旅游景区

围绕服务业发展“一个突破、五个提高”目标，培育一批拉动力强的消费增长点，重点加快旅游、金融、商贸物流业发展，不断增强消费对经济增长的基础作用。

加快推进旅游业大发展。立足做强山水旅游、提升乡村旅游、发展城市旅游，大力实施全市景区三年改造提升计划，加快推进上饶旅游集散中心、婺源国家乡村旅游度假实验区、灵山景区一期开发、神农源景区二期扩建、铅山古镇保护开发、大茅山温泉旅游度假区等重大新景区景点项目建设，尽快形成上饶旅游新的增长极。高标准规划、建设旅游公路、步道、服务区和高星级旅游饭店。深化品牌创建工作，加快灵山、神农源、云碧峰国家森林公园等创建国家4A级旅游景区步伐，启动龟峰创建国家5A级景区和鄱阳湖国家湿地公园、铜钹山、大鄣山景区创建国家级风景名胜区工作。深入挖掘上饶丰富的红色文化、书院文化、理学文化、稻作文化等历史文化资源，努力形成地方传统特色和现代文明和谐一体的上饶文化旅游。强化旅游市场营销，加强与周边地区在产品开发、联合促销、信息共享、客源互送等方面的协作配合，进一步提升上饶旅游的影响力和美誉度。加强旅游监管和服务质量保障体系建设，进一步提高行业管理和旅游标准化服务水平。力争全年接待游客突破5000万人次，旅游综合收入突破400亿元。

婺源县是首批中国旅游强县、全国旅游标准化示范县、国家乡村旅游度假实验区，旅游主要指标排在中国旅游强县第5位。三清山成功创建世界自然遗产、世界地质公园、国家5A级景区，成为全国第五个拥有这些殊荣的景区，排在全国旅游百强景区第22位。无论是婺源，还是三清山，都面临加快旅游业转型升级步伐、着力提升发展档次和水平的重大课题。今年，我们要把全力支持婺源建设江西第一、全国十强旅游县，全力支持三清山建设全国一流旅游景区作为旅游重点工作来抓。要加快重点项目建设，做精做细婺源旅游服务区、三清山金沙、枫林旅游服务区，完成婺源灵岩洞-景德镇瑶里、婺源段莘-浙源、清华-沱川等重点旅游公路建设，促进婺源国际大酒店、三清山国际度假大酒店年内进入五星级饭店评定程序；要狠抓品牌创建，确保婺源江湾创建国家5A级旅游景区获得成功，启动婺源汪口、篁岭创建国家4A级旅游景区，争取婺源汪口、理坑作为安徽皖南古村落世界文化遗产拓展项目进入世遗行列；要提升服务水平，帮扶三清山、婺源建设智慧城市、智慧导游、智慧酒店、智慧景区，发展电子门票、移动支付等，让游客体会到更便捷的服务；要培育旅游发展主体，鼓励三清山、婺源旅游集团以重组、兼并、收购等方式建立股份制旅游公司，争取早日上市。力争今年三清山接待游客700万人次、实现旅游综合收入50亿元，婺源接待游客1000万人次、实现旅游综合收入60亿元。

大力支持金融业做大做强。积极争取更多的银行、证券、保险等各类金融机构入驻我市，完成信州区联社、广丰县农合行、婺源县联社改制组建农村商业银行的工作，力争50%以上的县（市、区）设有村镇银行。支持小额贷款公司增资扩股，重点支持设立注册资金在1亿元以上的小额贷款公司；重点引导设立注册资金在5000万元以上、政府通过控股企业出资并参与管理的融资性担保机构。加快上饶金融商务区建设，推进金融产品和服务方式创新。促进银企合作，加大对重点产业、重点项目、小微企业、涉农企业、民生工程的资金支持力度，提高银企签约项目的履约率。力争鑫星农牧上市发行成功，加快耐普矿机上市步伐。完善对金融机构支持地方经济发展的考核激励机制，调动各金融机构的积极性。力争金融机构本外币存款余额新增260亿元，达到1646亿元；贷款余额新增150亿元，达到972亿元；银行业和资本市场等各项融资余额达到1260亿元。

大力推进商贸物流业发展。加快现代服务业集聚区建设，抓紧推进万年青建材商城、中国南方棉布批发市场二期、广丰红木文化创业产业园、婺源国际旅游商品城、万年国际珍珠城、鄱阳湖农资大市场、玉山旅游文化综合体等重大商贸物流项目建设，引导相关服务业企业向园区集聚。加大商贸流通业“2020”工程推进力度。深入实施“万村千乡”市场工程，推进农贸市场改造升级。大力发展电子商务、连锁经营、特许经营、大型超市、仓储式超市等新兴业态。

（四）加快推进新型城镇化，全力支持上、广、玉、铅、弋、横全面对接中心城市、全面融入中心城市，全力支持广丰、德兴跻身全国百强县

围绕“123”目标，充分挖掘城镇化对扩大内需、促进发展的巨大潜能。

加快重大交通和能源项目建设。推进上饶三清山机场、合福高铁、杭长客专、320国道上饶城区段改建、德上高速三清山和怀玉山连接线、沪昆高速上饶经开区互通、广丰至上饶火车站公路等项目建设。逐步实现干线公路大部分达到二级及以上等级水平，农村公路50%县道达三级公路技术标准、80%的乡道达四级公路技术标准的目标。开工建设九景衢铁路、鄱阳湖旅游公路、宁德港上饶码头。开工建设黄金埠电厂二期工程，续建“西气东输”二线和中石化江西成品油管道二期工程。实施中心城市电力管网改造。加快推进上饶至万年高速公路、上饶至铅山快速通道、鄱阳港务区、上饶货场西迁、皖赣铁路电气化改造、上饶赣东火力发电厂、经开区分布式能源站等重大项目前期工作。

完善城镇规划体系。加快完成中心城市各片区控制性详细规划编制，全面完成《上饶都市区“1+5”规划》、《上饶市综合交通枢纽工程及高铁核心区城市设计》等重大规划编制。全面完成县（市）总体规划修编，加快推进县城生态系统规划、近期建设规划、市政综合管网规划、综合交通规划及示范镇控制性详细规划编制，力争县城和示范镇控规覆盖率达到80%以上。

推进中心城市建设。重点实施十大工程：一是全线贯通南北向长30公里的上饶大道（含机场大道、上饶南大道、上饶北大道、灵山大道）。二是全线贯通东西向长30公里的凤凰大道（含凤凰大道东延伸和西延伸）。三是推进老火车站城市综合体建设。四是推进广信大厦建设。五是推进上饶市三甲医院及康复中心等医疗卫生设施建设。六是实施三江水系、信江南岸河堤、云碧峰入口等特色景观改造。七是推进中心城市保障性安居工程建设。八是推进上饶师范迁建。九是加快市政、环卫、园林基础设施改造建设。十是完成城市供水水源引水工程。

上饶、广丰、玉山、铅山、弋阳、横峰六县，都在上饶中心城区半小时经济圈内，基本与上饶中心城区连为一体。今年，要把全力支持上、广、玉、铅、弋、横全面对接中心城市、全面融入中心城市作为加快城镇化的重点工作来抓。要按照“1+5”都市区规划要求，加快320国道的改造升级及客运枢纽建设，实现道路交通的区域快速对接；要加强区域产业分工协作，中心城市、上、广、玉、铅、弋、横要以有色金属、新能源、机电光学和新型建材四大产业为主导，形成工业、农业、旅游业、服务业齐头并进的合作联盟，加快构建“1+5”都市区区域生态产业链；要加大“1+5”都市区发展政策支持力度，探索建立城镇空间、产业区划、基础设施协调对接机制，积极打造“生态河谷绿都”。

提升城市管理水平。进一步创新城市管理体制机制，建成并运行中心城市数字化城管系统。加大市容市貌整治力度，逐步提高中心城市环卫作业机械化和市场化水平。实施城市综合治污工程，加大城市地下管网建设和改造力度。优化城市交通环境，大力整治交通秩序，合理布局停车场所，切实治理好老城市拥堵问题。坚持公交优先，进一步理顺公交管理体制，加大公交投入，完善公交设施，更新公交车辆60台。

大力发展县域经济。深入推进扩权强县、兴县强镇试点改革，引导更多发展要素向县城倾斜，不断激发县域经济发展的内生动力。完善提升县城功能，积极推进产业基础好、经济实力强的县城加快发展。支持万年县县域经济发展先行先试。以中心镇和示范镇为重点，加大小城镇建设力度，力争县县财政收入过10亿元，其中广丰、德兴财政收入过30亿元，新增玉山、余干生产总值超100亿元。

广丰、德兴资源丰富，经济基础好、经济规模大、经济实力强，在全国县域经济中排位200名左右，具有巨大的发展潜力。今年，我们要把全力支持广丰、德兴跻身全国百强县作为县域经济发展的重点来扶持。要发挥资源优势，着力打造特色产业；要加大主攻工业力度，着力打造“千亿元”工业园区；要统筹城乡协调发展，迅速做大经济总量；要制定相关政策，支持广丰、德兴打造经济增长极。力争通过努力，每年跃上一个新台阶。

大力推进城乡一体化发展。加大城乡统筹力度，形成以工促农、以城带乡、工农互惠、城乡一体的新型城乡、工农关系，加快形成“体制统一、规划统筹、资源共享、利益共得”的城乡一体化发展格局。深化户籍制度改革，抓紧制定必要的配套政策，畅通农民转变居民的渠道。深化行政区划改革，加大撤销乡（镇）改为街道办事处、撤销村委会改为居委会的力度，让农民成为城镇居民。推进公共服务均等化，切实解决好进城农民的就业、安居、子女就学、社会保障等问题，实现农业转移人口市民化。

（五）加快推进改革开放，全面建设面向东南沿海对接合作示范区

坚持用改革的办法破解发展中的难题，全面深化各项改革，坚定不移扩大开放。

大力实施创新驱动。着力构建以企业为主体、市场为导向、产学研相结合的技术创新体系，加速科研成果的产业化。积极培育申报省战略性新兴产业研究开发重点项目、国家科技型中小企业技术创新基金及国家科技富民强县项目。加快新技术、新产品、新工艺研发应用，加强技术集成和商业模式创新。支持促进高新技术企业快速发展，力争新增5家高新技术企业。加强知识产权保护，体现科技原创价值。实施质量兴市战略，推进品牌和标准化建设。

深化重点领域改革。完善国有资产监管体制，巩固国有工业企业和非工口7个系统国有企业改革成果。完善基本药物制度和基层医疗卫生机构运行机制，深化基层卫生机构综合改革。继续推进事业单位分类改革。巩固文化体制改革成果。深化财税金融体制改革，完善国库集中收付制度。扎实推进基层农技推广体系改革。力争年底前全面完成国有林场改革。稳妥推进其他各项改革。

提高开放型经济水平。依托我市产业优势和产业集聚区平台，积极引进外商投资功能性机构及区域总部。深度挖掘合作潜力，不断拓展与央企、民企对接和东南沿海对接合作的深度和广度。力争新引进省外5000万元项目资金330亿元，实际利用外资达到7.2亿美元。大力培育生产型出口龙头企业，依托上饶茶叶国家级外贸转型升级专业性示范基地和信州光学国家级科技兴贸创新示范基地，推动外贸出口转型升级，实现外贸出口27亿美元。加快南昌海关上饶办事处升格为上饶海关步伐，巩固和发展上饶“无水港”、上饶至宁波 “五定班列”开放平台优势，推动“五定班列”沿沪昆线向西延伸。

支持全民创业。进一步完善和落实促进非公经济发展的各项政策措施，鼓励支持有创业愿望的各类人才发展实体经济，鼓励支持非公经济进入基础产业、基础设施、社会事业、金融等领域。加快创业园区、小微企业创业园等创业平台建设，完善中小企业社会化服务体系，促进非公经济和中小微企业快速健康发展。力争全市非公经济增加值占生产总值的比重达到66%以上。

今年，我们要积极争取省委、省政府的支持，以建设面向东南沿海对接合作示范区为抓手，按照“立足鄱湖，对接东南，全面融入，超常发展”总体思路，进一步深化与东南沿海地区对接合作，全方位深化改革、扩大开放。重点是推进思想观念、体制机制、产业技术、人才科技、生产要素、交通设施、教育文化等方面与东南沿海的全面对接，实现资源优化配置，形成与东南沿海地区产业联动、优势互补、分工协作、共同发展的格局。

（六）加快推进生态文明建设，全力支持上饶县建设再生资源循环经济产业园

坚持节约资源和保护环境的基本国策，着力推进绿色发展、循环发展、低碳发展，不断巩固和提升生态优势，努力把上饶建成“秀美江西”的名片、“美丽中国”的亮点。

深入推进生态建设。大力开展造林绿化，加强森林资源管护，加快实施“森林城乡、绿色通道”工程，完成35万亩造林任务，建成森林乡镇50个、森林村庄350个、生态文化示范基地10个，提高森林覆盖率和城市建成区绿化覆盖率。启动国家级森林城市创建工作，加强自然保护区、森林公园和湿地公园建设管理，开展生态县（市）、生态工业园区、生态乡（镇）、生态村创建活动，年内新增省级生态乡（镇）、生态村各10个，市级生态村50个。

不断加强环境保护。强化环境整治，严格落实环保目标责任制，加强重点区域、重点行业、重点企业污染防治，加快德兴、弋阳、铅山、横峰等国控、省控重金属污染防治重点区域的环境整治，坚决关停取缔 “十五小”、“新五小”等违法排污企业。加强水环境保护，在信江、饶河上游沿岸1公里范围内人口较为集中的村镇，规划建设一批生活污水和生活垃圾处理设施。加强大坳水库水环境保护，完成大坳库区水源保护地划定和整治工作，确保饮用水源安全。

切实抓好节能减排。落实国家能源消费强度和总量双控制要求，突出抓好工业、建筑、交通、公共机构等领域节能，推行节能技术，淘汰落后产能，鼓励清洁生产。加快污水处理设施建设，加快推进全市工业园区污水处理厂和德兴、铅山等7个生活垃圾无害化处理项目建设，完善城镇生活污水管网建设，确保全市11个污水处理厂正常运行，确保完成节能减排各项目标任务。

去年，我市被列入全国第三批再生资源回收体系建设试点城市，前期工作已经启动，再生资源循环经济产业园项目选址在上饶县茶亭工业园区。今年，要全力支持上饶县建设再生资源循环经济产业园。要坚持走高起点规划、高标准建设、高强度投入的路子，把园区建成生态环保、全国一流的再生资源循环经济产业园；要加快园区基础设施建设，为企业尽快入园打好基础；要招大引强，引进有影响、有实力、有经验、有品牌的全国知名龙头企业进入园区；要搞好以中心城市为重心，覆盖“半小时经济圈"7个县（区）、并兼顾其他5个县（市）的标准化再生资源回收网络建设。力争到年底，完成城市90%社区回收点建设和再生资源回收网点的整合、改造，完成乡镇回收站、回收中转站网络建设，形成统一规范的再生资源回收网络体系。

（七）加快推进社会事业发展，全力支持上饶师范学院创建综合性大学，全力支持上饶卫校、上饶师范创建大专院校

坚持把惠民生、促和谐放在政府工作的首位，不断扩大和改善公共服务，加快发展社会事业，努力提升人民群众的幸福感和满意度。

稳定和扩大就业。实施就业优先战略和更加积极的就业政策，多渠道多形式开发就业岗位，促进创业带动就业，新增城镇就业4.7万人。做好以高校毕业生为重点的青年就业和农村转移劳动力、城镇困难人员、退役军人就业工作。加大就业创业扶持力度，新增小额担保贷款6亿元。完善社会保障体系。全面建成覆盖城乡居民的社会保障体系。力争城镇职工基本养老保险参保人数达到72.6万人，城乡居民养老保险参保人数达到236万人，失业保险参保人数达到29万人，失地农民参加养老保险人数达到5万人。城镇基本医疗保险参保人数达到124.9万人，工伤保险参保人数达到33.7万人，残疾人康复1.73万人。

提高社会救助水平。加快推进城镇居民家庭收入核对中心建设，城市低保、农村低保和城镇“三无”特困群众月平均保障标准分别提高到400元、200元和500元，农村五保户集中和分散供养月标准分别提高到245元和220元。加大社会救助力度，将儿童白血病、先天性心脏病、尿毒症、重性精神病等20类疾病纳入保障范围。成立市级减灾备灾中心，提高应对自然灾害救助能力。加快发展老龄事业，全面建立80周岁以上高龄老人津贴制度。

加大扶贫开发力度。继续实施整村推进、以工代赈和移民扶贫搬迁工程，搬迁移民6000人。加大对缺乏劳动能力贫困人口的救济和救助力度。实施教育扶贫，阻断贫困代际传递。加快保障性安居工程建设。认真贯彻落实国家调控房地产市场的各项政策措施，促进房地产业规范有序健康发展。完善经济适用住房、廉租住房、公租住房“三房合一、租售并举”制度。加大利用住房公积金支持保障性住房建设试点力度，新增保障性住房1万套，全年建设保障性住房达到5万套。

大力加强文化建设。积极培育和践行社会主义核心价值观。发展新型文化业态，促进文化产业规模化、集约化、专业化发展，提升文化软实力。深入实施文化惠民、基础文化设施提升和基层文物保护工程。实施村村通广播电视工程703个，更新农家书屋260个。加强农村和社区文化建设，积极开展群众性文化活动。强化网络社会管理，推进网络依法规范有序运行。大力繁荣文化市场，鼓励各类艺术团体开展丰富多彩的文化活动，博物馆、纪念馆、图书馆、文化馆(站)等继续向群众免费开放。

协调发展社会事业。坚持教育优先发展，强化教育投入机制，统筹发展各类教育。做好进城务工人员子女入学和留守儿童教育管理工作。推进医药卫生事业发展，加强基本公共卫生均等化服务。推进鄱阳湖南岸片区血吸虫病综合防治示范区建设。推进农村安全饮水工程，实施农村自来水工程。广泛开展全民健身运动，办好第三届全市运动会，提高竞技运动水平。认真抓好人口计生工作，稳定低生育水平，持续综合治理出生人口性别比问题，促进人口长期均衡发展。实施遗体火化三项基本费用免费制度。进一步做好统计、物价、防震减灾、民族宗教、外事侨务和对台等工作，加强国防教育、人防民防、民兵预备役建设，巩固扩大双拥共建成果，建成上饶一级军供站。支持各民主党派、工商联、无党派和工会、共青团、妇联、社联、科协、残联的工作。发展高等教育事业是建设宜居宜业宜游现代化区域中心城市的必然要求，是上饶高等教育事业改革发展的现实需要。

今年，我们要着手开展全力支持上饶师范学院创建综合性大学，全力支持上饶卫校、上饶师范创建大专院校工作。一是启动建设综合性上饶大学工作，力争通过5年左右时间，在上饶师范学院的基础上，建成集文、理、教、史、法、工、农、经、管、医等学科的综合性上饶大学。二是在上饶卫校的基础上加快上饶医学高等专科学校建设。上饶卫校申报高等专科学校去年底已基本通过省教育厅组织的专家组评估，要按照专家组的意见尽快完善建设条件。三是加快推进上饶幼儿师范高等专科学校建设。以上饶师范学院小教分院（上饶师范）、万年师范为基础设立上饶幼儿师范高等专科学校已列入《江西省“十二五"期间高等学校设置规划》，目前要加快学校的搬迁建设，确保于2015年7月份前顺利通过省教育厅的评估验收。

（八）加快推进社会管理创新，促进社会和谐稳定

坚持把创新社会管理作为统筹经济社会发展的重要举措和维护社会稳定的根本要求，进一步加强和创新社会管理，不断提高社会凝聚力。

加强基层基础建设。全面落实“党委领导、政府负责、社会协同、公众参与、法治保障”的要求，推动社会建设和管理重心下移，整合各种资源，不断提高基层社会管理能力。扎实推进社区规范化建设和网格化管理，加快改善社区办公和服务用房条件，强化社区自治和服务保障能力。

加强和改进新形势下群众工作。推进信访工作创新，积极落实领导干部定期接待群众来访和部门联合接访制度，大力推广“万年模式”，完善人民调解、行政调解、司法调解联动的工作体系，构建联系群众、服务群众的长效机制，加大矛盾纠纷排查化解力度。建立健全重大决策社会稳定风险评估机制，不断完善涉及群众切身利益的重大决策和重大项目社会公示、听证等制度。促进社会公平正义，畅通和规范群众诉求表达、利益协调、权益保障渠道。

加强公共安全体系建设。严格落实安全生产责任制，加大交通运输、矿山、建筑施工、烟花爆竹、危险化学品等重点领域隐患排查整治力度，推进煤矿企业兼并重组，有效防范和坚决遏制重特大事故发生。加强人员密集性场所、棚户区危旧房等重点领域消防安全。加强食品药品安全工作，推进食品生产经营企业诚信体系建设，不断提高食品卫生和药品安全保障水平。大力开展“六五”普法教育，深入推进平安上饶建设，加快完善覆盖城乡的社会治安防控体系。加强流动人口和特殊人群服务管理，严厉打击各类违法犯罪活动，全力维护社会稳定。加强应急管理工作，重点完善应急管理体系和应急救援队伍建设，提高应对自然灾害、事故灾难、公共卫生事件和社会安全事件的应急处置能力。

（九）以全面建成小康社会为己任，着力加强政府自身建设

全面建成小康社会，是党和人民赋予我们的历史使命。与全国同步建成小康社会指标相比，我们还有不小差距。政府的责任就是要尽快缩小这些差距，给人民交上一份满意的答卷。

要始终保持奋发有为的精神状态。扎实推进学习型政府建设，用党的十八大精神和中国特色社会主义理论武装头脑、指导实践、推动工作。坚持把解放思想作为加快发展的原动力，注重运用改革创新的办法解决发展中的困难和矛盾，不断增强预见性、把握主动性、提高针对性。瞄准既定目标，进一步增强忧患意识、创新意识、宗旨意识和使命意识，勇于开拓、敢于担当，干好每一天、走好每一步。

要始终坚持真抓实干的工作作风。严格落实中央关于改进工作作风、密切联系群众的八项规定。要改进调查研究，轻车简从，深入基层，深入实际，既要到工作形势好的地方去总结经验，更要到困难和矛盾集中、群众意见多的地方去商量解决问题。要切实改进会风，坚持少发文、少开会，严格控制数量，严格控制规模，严格领导出席，严格控制经费，严格审批程序。必须召开的会议，要安排紧凑，简化程序，讲实话、讲短话、讲管用的话。要密切联系群众，同群众坐在同一条板凳上，“当面锣、对面鼓”，与群众真心交流、真情沟通、真诚相待。要真抓实干。政府的职责就是干事。决策一旦确定，各级政府和部门就要闻风而动、雷厉风行，一以贯之、一抓到底。要强化政务督查，抓住不落实的人、严查不落实的事，确保政令畅通。

要始终秉持依法行政的履职理念。政府的各项工作，只有依法依规，才能经得起人民的检验、历史的检验。各级政府要主动接受人大法律监督，虚心接受政协民主监督,诚恳接受社会监督，广泛接受群众监督，让权力在阳光下运行。要及时办理人大议案、代表建议和政协提案，扩大沟通率、主攻解决率、提升满意率。要认真听取各民主党派、工商联、无党派人士及各人民团体的意见，充分采纳社会各界的真知灼见。要进一步加强电子政务建设，推进政务公开，切实提高政府工作透明度和公众参与度。

要始终维护清正廉洁的良好形象。坚持用制度管权管事管人，完善风险岗位廉能管理，健全政府重大投资项目公示制和责任追究制，规范招投标管理。加强对土地管理、工程建设、征地拆迁、住房保障、环境保护等重点领域的行政监察和审计监督，坚决纠正损害群众利益的不正之风，着力解决发生在群众身边的腐败问题。深入推进节约型机关建设，严格控制“三公”经费。全体政府工作人员都要坚定信念、牢记使命、自警自律，不越“红线”、不破“底线”、不触“高压线”，以优良的政风赢得群众的信任和支持。

各位代表！新的目标催人奋进，新的征程已经开启。让我们紧密团结在以习近平同志为总书记的党中央周围，在中共上饶市委的坚强领导下，高举中国特色社会主义伟大旗帜，紧紧依靠全市人民，解放思想，振奋精神，凝心聚力，攻坚克难，为把上饶建设成为宜居宜业宜游的现代化区域中心城市而努力奋斗！
